# Correlation of MicroRNA-16, MicroRNA-21 and MicroRNA-101 Expression with Cyclooxygenase-2 Expression and Angiogenic Factors in Cirrhotic and Noncirrhotic Human Hepatocellular Carcinoma

**DOI:** 10.1371/journal.pone.0095826

**Published:** 2014-04-23

**Authors:** Wenjiao Zeng, Anke van den Berg, Sippie Huitema, Annette S. H. Gouw, Grietje Molema, Koert P. de Jong

**Affiliations:** 1 Department of Pathology & Medical Biology, University Medical Center Groningen, University of Groningen, Groningen, The Netherlands; 2 Department of Pathology & Medical Biology. Medical Biology Section, University Medical Center Groningen, University of Groningen, Groningen, The Netherlands; 3 Department of Hepato-Pancreato-Biliary Surgery & Liver Transplantation, University Medical Center Groningen, University of Groningen, Groningen, The Netherlands; National Institutes of Health, United States of America

## Abstract

**Background:**

Hepatocellular carcinoma (HCC) is a classical example of inflammation-linked cancer and is characterized by hypervascularity suggesting rich angiogenesis. Cycloxygenase-2 (COX-2) is a potent mediator of inflammation and is considered to upregulate angiogenesis. The aims of the study are (1) to analyze expression of Cox-2 mRNA, Cox-2 protein, miR-16, miR-21 and miR-101 in HCC and adjacent liver parenchyma in cirrhotic and noncirrhotic liver, (2) to investigate the relation between COX-2 expression, miR-21 expression and angiogenic factors in these tissues and (3) to investigate the association between miR-16 and miR-101 and COX-2 expression.

**Methods:**

Tissue samples of HCC and adjacent liver parenchyma of 21 noncirrhotic livers and 20 cirrhotic livers were analyzed for COX-2 expression at the mRNA level (qRT-PCR) and at the protein level by Western blot and immunohistochemistry. Gene expression of VEGFA, VEGFR1, VEGFR2, Ang-1, Ang-2 and Tie-2 were correlated with COX-2 levels. miR-16, miR-21 and miR-101 gene expression levels were quantified in HCC tumor tissue.

**Results:**

COX-2 mRNA and protein levels were lower in HCC as compared to adjacent liver parenchyma both in cirrhotic and noncirrhotic liver. COX-2 protein localized mainly in vascular and sinusoidal endothelial cells and in Kupffer cells. At the mRNA level but not at the protein level, COX-2 correlated with mRNA levels of angiogenic factors VEGFR1, Ang-1, and Tie2. miR-21 expression was higher in cirrhotic tissues versus noncirrhotic tissues. MiR-101 expression was lower in cirrhotic versus noncirrhotic adjacent liver parenchyma. None of the miRNAs correlelated with COX-2 expression. miR-21 correlated negatively with Tie-2 receptor in adjacent liver parenchyma.

**Conclusions:**

In human HCC, COX-2 mRNA but not COX-2 protein levels are associated with expression levels of angiogenic factors. MiR-21 levels are not associated with angiogenic molecules. MiR-16 and miR-101 levels do not correlate with COX-2 mRNA and protein levels.

## Introduction

More than 80% of hepatocellular carcinomas (HCC) are associated with chronic infection caused by hepatitis B or C virus [1]. In well-developed countries with a high incidence of obesity and diabetes mellitus the risk of developing HCC rises proportionally with increasing body mass index and the duration of diabetes [2], [3]. These conditions are frequently associated with non-alcoholic steatohepatitis (NASH) [4], [5]. The common denominator in viral hepatitis associated liver diseases and NASH is presumed to be the chronic inflammation leading to fibrosis and cirrhosis and ultimately to HCC. HCC is thus one of the classical examples of inflammation-linked cancer [6]. The cyclooxygenase-2 (COX-2)- prostanoid pathway plays a pivotal role in inflammation and in the pathophysiology of liver diseases like cirrhosis and HCC [7]. COX-2 is an inducible immediate-early gene originally found to be induced by various stimuli including mitogens, cytokines and growth factors [8]. COX-2 might favor tumor growth by various mechanisms including stimulation of angiogenesis, evasion of apoptosis and propensity to metastatic behavior and invasion. COX-2 inhibitors can block these mechanisms by various pathways and selective COX-2 inhibitors have been evaluated for their effect on HCC cell growth and invasion using animal models of hepatocarcinogenesis [9], [10].

HCC can also arise in noncirrhotic livers with normal histology and no signs of inflammation or viral hepatitis. A common characteristic of both types of HCC is hypervascularity. The occurrence of both types of HCC in humans offers a unique opportunity to study the possible relation between inflammation and angiogenesis, in which angiogenic characteristics can be analyzed in the presence (cirrhotic HCC) or absence (noncirrhotic HCC) of chronic inflammation. Unraveling of the underlying mechanisms might lead to more efficacious treatment modalities both for (chemo)prevention and possibly also for treatment of already established HCC.

The aim of the present study is to investigate the correlation of COX-2 expression with the expression of angiogenic factors in human HCC in cirrhotic and noncirrhotic livers. We investigated gene and protein expression levels of COX-2 and correlated these to gene expression levels of VEGF-A, VEGFR-1 (Flt-1) and VEGFR-2 (KDR), and Angiopoietin (Ang)-1, Ang-2, and their receptor Tie-2 in HCC, adjacent liver parenchyma and normal liver parenchyma. The cellular localization of COX-2 was studied by immunohistochemistry. Several papers highlighted the role of microRNAs (miRNAs) in the control of COX-2 transcription. MiRNAs are small noncoding RNAs that control gene expression at the posttranscriptional level. Their role in HCC has recently been reviewed [11]. Especially miR-21, miR-101 and miR-16 seem to be relevant for the present study. MiR-21 is an oncogene with high expression levels in various cancers including HCC. Additionally, miR-21 is strongly related to angiogenesis [12], [13]. Furthermore, accumulating evidence links miR-21 to inflammation [14]–[16] including the necroinflammation in the liver which ultimately results in liver cirrhosis [17]. In contrast, miR-101 is considered a tumor suppressor gene and its expression was inversely correlated with COX-2 expression in colon cancer and gastric cancer [18], [19]. Another miRNA shown to inhibit COX-2 expression is miR-16. In several hepatoma cell lines an inverse relation between miR-16 and COX-2 was found [20].The same authors describe a negative correlation between COX-2 protein and miR-16 expression in seven human HCC and paired non-tumoral liver biopsies.

The study aimed at analysing (1) the correlation between COX-2 expression and angiogenic factors, (2) the correlation between COX-2 expression and miR-101/miR-16 and (3) the correlation between angiogenic factors and miR-21 in cirrhotic and non-cirrhotic HCC, both in tumor tissue and adjacent liver parenchyma.

## Patients and Methods

All (anonymised) tissue samples were processed according to national guidelines. According to the Dutch law no approval of an Institutional Review Board is necessary for investigations using archival materials of previously resected tissue specimens. Tissue samples of 41 HCC patients were included; 21 noncirrhotic HCC obtained from partial liver resection specimens and 20 cirrhotic HCC obtained from liver explants after liver transplantation. Clinicopathological characteristics of the patients are summarised in Table 1. A sample of adjacent, non-tumorous liver tissue was also included in the study. None of the HCC patients underwent previous treatments like local ablation, chemoembolisation or chemotherapy.

**Table 1:** Clinicopathological characteristics of the cirrhotic and noncirrhotic patient group.

Clinicopathological variable	Noncirrhotic	Cirrhotic	
	n = 21	n = 20	
**Sex** (male/female)	13/8	15/5	
**Underlying liver disease**	Absent	HCV	4
		Alcoholic liver disease	3
		cryptogenic	3
		hemochromatosis	2
		metabolic disease 1	5
		various	3
**Age** median (IQR)	56.3 (27.5)	58.6 (10.8)	
**α-fetoprotein** (µg/L; median (IQR))	7.0 (428)	17 (44)	
**Largest HCC diameter** (cm; median (IQR))	13.0 (10)	3.5 (2)	
**Number of HCC nodules** median (IQR)	1 (0)	2 (6)	

1 α1-antitrypsin deficiency (n = 2); glycogenosis type III (n = 1); tyrosinemia (n = 2).

Samples from histological normal livers (n = 8), obtained from surplus donor liver or partial liver resection for benign disorders, were used as a reference and not for statistical comparisons. HCCs were graded according to the Edmonson classification and COX-2 mRNA level and protein expression was compared among the three classes.

### RNA extraction and quantitative reverse transcription polymerase chain reaction (qRT-PCR)

Quantitative RT-PCR was performed as described previously [21]. Intron overlapping primer sets and minor groove binder probes were purchased as Assay-on-Demand from Applied Biosystems (Nieuwekerk a/d IJssel, The Netherlands): housekeeping gene GAPDH (assay ID Hs99999905_m1), COX-2 (assay ID Hs00153133_m1), VEGF (Hs00173626_m1), VEGFR-1 (Hs00176573_m1), VEGFR-2 (Hs00176676_m1), Tie2 (assay ID Hs00176096_ml), Ang-1 (assay ID Hs00181613_ml), Ang-2 (assay ID Hs00169867_ml).

### MiRNA analysis

Expression levels of miR-16, miR-21 and miR-101 were determined by qRT-PCR using RNU49 as housekeeping gene with miRNA qRT-PCR assays (Applied Biosystems, Foster City, USA) as described previously [22]. Reverse transcription (RT) primers specific for a miRNA and RNU49 were multiplexed in 15 µl RT reactions containing 1 µl of each RT primer. The miRNA levels were normalized to the RNU49 levels. Mean cycle threshold (C_t_) values for all genes were quantified with the SDS software (version 2.1). Relative expression levels were calculated as 2^−ΔCt^.

### Western blot

Western blotting was performed as described previously using mouse anti-COX-2, 1∶500 (BD Biosciences Pharmingen, #610204) [21]. Quantification of the signals detected was done by relating COX-2 to β-actin control. The bands of COX-2 protein located at about 70 kDa as expected according to the datasheet of the antibody.

### Immunohistochemical examination of COX-2 expression

Four µm tissue sections were deparaffinized and treated with 0.08% H_2_O_2_ for 30 min to block endogenous peroxidase. Slides were incubated with mouse anti-COX-2 (BD Biosciences Pharmingen, #610204) 1∶50, diluted in 1%BSA/PBS, at 4°C overnight followed by incubation with horseradish peroxidase-conjugated rabbit anti-mouse Ig and goat anti-rabbit Ig (DAKO), both 1∶100 diluted in 1%BSA/1% albumin/PBS. Diaminobenzidin was used to develop the staining reaction and nuclear counterstaining was performed with haematoxylin. A sample of a human colonic adenocarcinoma was included as a positive control.

### Correlation between COX-2 protein and mRNA level

Pearson correlation coefficients were calculated for COX-2 mRNA and COX-2 protein separately for HCC and liver parenchyma. We calculated the COX-2 protein:COX-2 mRNA ratio for each individual case; this method has been used for identification of the strength of correlation of mRNA expression and protein level of individual genes by Pradet et al [23].

### Correlation of COX-2 and miR-21 with Ang-2:Ang1 ratio

Although in many tumors both Ang-1 and Ang-2 are elevated, the angiogenic state of hypervascular tumors is characterized by an overexpression of Ang-2 in relation to Ang-1 thus creating a shift in the Ang-2:Ang-1 ratio in favor of Ang-2 [24]. A higher Ang2:Ang1 ratio was also encountered in human HCC [24]. Therefore we analyzed the correlation between COX-2, miR-21and the Ang-2:Ang-1 gene expression ratio.

### Statistical analysis

Quantitative data were expressed as median and interquartile range (IQR). Expression of COX-2 mRNA, COX-2 protein, miR-16, miR-21 and miR-101 were compared using a two-tailed Mann-Whitney-U test (non-related samples) in cirrhotic versus noncirrhotic liver of HCC and adjacent parenchyma separately. The Wilcoxon test (related samples) was used for comparison of HCC with adjacent parenchyma in cirrhotic and noncirrhotic liver separately. For correlation statistics, values were normalised by log (base 10) transformation. Pearson correlation coefficients were calculated between COX-2 and the angiogenic factors, between miR-21 and the angiogenic factors and between miR-101 and miR-16 and both COX-2 mRNA and COX-2 protein. A p-value <0.05 was considered significant.

## Results

### COX-2 expression in HCC and adjacent liver parenchyma in cirrhotic and noncirrhotic liver

**Figure 1:**
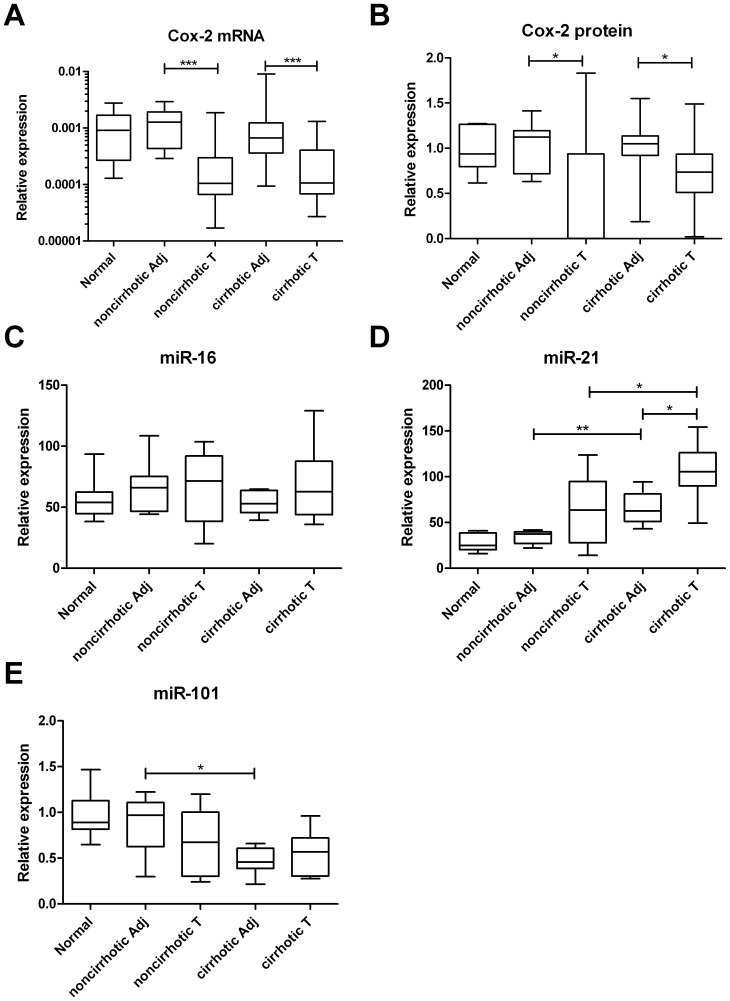
Plots representing relative expression of COX-2 mRNA (A), COX-2 protein (B), miR-16 (C), miR-21 (D) and miR-101 (E) in in normal liver, HCC tumor tissue (T) and tumor-adjacent parenchyma (Ad) in noncirrhotic and in cirrhotic liver. Median values and 25^th^–75^th^ percentiles are shown. P-values: *<0.05; **<0.01; ***<0.001.

**Figure 2:**
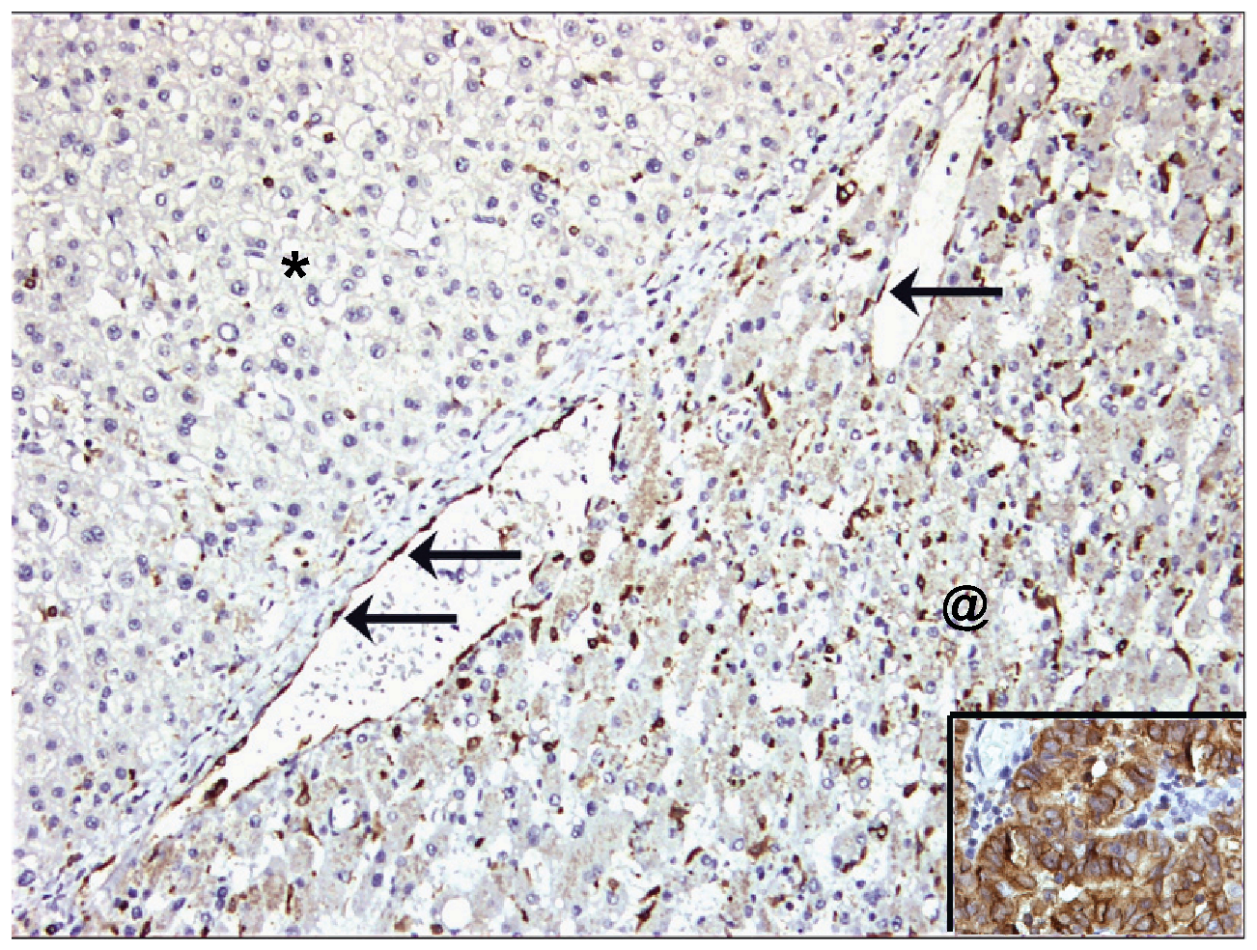
COX-2 immunostaining on HCC (upper left, marked with *) and adjacent liver parenchyma (lower right, marked with @). COX-2 expression is present on endothelial cells (arrows) at the interface between HCC and non-tumorous liver parenchyma. In the liver parenchyma COX-2 expression is mainly present in Kupffer cells and some inflammatory cells. Note the absence of staining in hepatocytes and tumor cells. The insert (lower right hand corner) shows COX-2 immunostaining on coloncarcinoma as a positive control.

HCC tumor tissue in both cirrhotic and noncirrhotic liver demonstrated a lower COX-2 expression compared to adjacent parenchyma, both at the mRNA (Figure 1A) and protein level (Figure 1B). Median COX-2 mRNA level in the tumor in noncirrhotic liver was about tenfold lower than in tumor-adjacent liver parenchyma (p<0.001). In cirrhotic liver, HCC tumor had a six-fold lower COX-2 mRNA content than adjacent parenchyma (p<0.001). Protein levels of COX-2 were approximately 370 fold lower in HCC in noncirrhotic liver as compared to adjacent liver parenchyma (p = 0.033). In cirrhotic liver, HCC tumor had a 30% lower COX-2 protein level (p = 0.020) than adjacent parenchyma. A comparison between COX-2 mRNA and COX-2 protein expression in cirrhotic versus noncirrhotic HCC tumor tissue revealed no differences (Figures 1A and 1B). COX-2 staining in HCC was mainly found in endothelial cells lining the sinusoid-like spaces in the tumor but less conspicuous than in vascular endothelial cells at the interface between tumor and adjacent liver parenchyma. (Figure 2) As a positive control of COX-2 immunohistochemical staining we included a colon carcinoma sample which was strongly positive (Figure 2 insert). No difference was found in COX-2 mRNA level and protein expression between tumors with Edmonson grade 1, grade 2 or grade 3 (p>0.35).

COX-2 mRNA and COX-2 protein expression in tumor-adjacent liver parenchyma was comparable in cirrhotic and in noncirrhotic liver and in the same range as normal liver parenchyma (Figures 1A and 1B). COX-2 staining was observed in both vascular and sinusoidal endothelial cells as well as in Kupffer cells in normal liver and in parenchyma adjacent to HCC. No expression was found in hepatocytes or in cholangiocytes (Figure 2).

**Figure 3:**
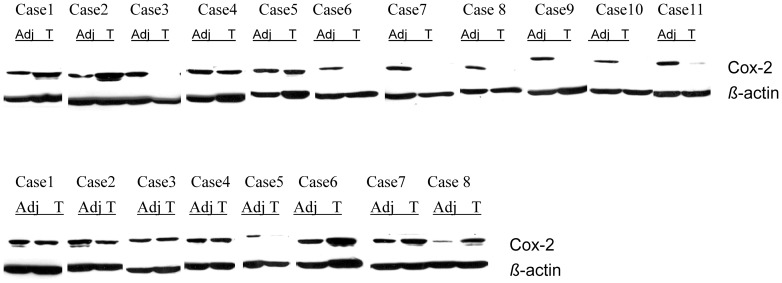
Example of representative cases analysed in this study showing Western blot of COX-2 in matched pairs of 11 noncirrhotic (upper panel) and 8 cirrhotic (lower panel) samples of HCC and tumor-adjacent liver parenchyma. (Adj: tumor-adjacent liver parenchyma; T: tumor (HCC)).

Although median COX-2 expression in HCC was less than in the surrounding parenchyma, individual cases demonstrated variable results as can also be seen in representative Western blots of COX-2 (Figure 3).

### Correlation of COX-2 gene expression and protein level with angiogenic factors in HCC tumor tissue

Of the six analysed angiogenic factors, VEGFR1, Ang-1 and Tie-2 revealed a significant correlation with COX-2 mRNA levels in HCC tumor tissue both in noncirrhotic and cirrhotic liver (Table 2). Additionally, a correlation between COX-2 mRNA and VEGFR2 mRNA was observed in HCC in noncirrhotic liver only (r = .78, p<0.001). No significant correlations were obtained between the angiogenic factors and COX-2 protein in HCC tumor tissue. Moreover, also the Ang-2:Ang-1 gene expression ratio did not show any correlation with COX-2 expression (Table 2).

**Table 2:** Correlation between COX-2 mRNA and protein level with mRNA level of various angiogenic factors.

	Noncirrhotic liver								Cirrhotic liver							
mRNA level angiogenic factor	COX-2 mRNA				COX-2 protein				COX-2 mRNA				COX-2 protein			
	tumor		adjacent		tumor		adjacent		tumor		adjacent		tumor		adjacent	
	r	p	r	p	r	p	r	p	r	p	r	p	r	p	r	p
VEGFA	.39	.08	.09	.79	−.57	.07	.05	.88	.26	.21	.20	.36	−05	.81	.11	.62
VEGFR1	**.86.**	**.001**	−.24	.49	−.48	.13	.54	.09	**.48**	**.02**	.08	.71	.09	.70	.06	.78
VEGFR2	**.78**	**.001**	.37	.26	−.47	.14	−.27	.42	.16	.44	−.05	.81	−.12	.60	−.38	.09
Ang-1	**.51**	**.02**	.15	.67	−.13	.71	−.23	.49	**.51**	**.01**	**.42**	**.04**	.22	.32	−.02	.95
Ang-2	.13	.58	−.12	.73	−.41	.21	.21	.54	.08	.72	.24	.27	.11	.63	.08	.71
Tie-2	**.82**	**.001**	−.22	.51	−.42	.20	−.42	.20	**.68**	**.001**	.21	.35	.18	.42	−.08	.73
Ang-2:Ang-1	−.40	.07	−.18	.60	−.3	.37	.34	.31	−.36	.08	.24	.27	−.14	.53	.08	.71

Pearson r (r) and p-value (p) are presented in tumor and adjacent cirrhotic and noncirrhotic liver separately. Statistically significant correlations are shown in bold. Abbreviations: Ang-1/2: angiopoietin-1/2; Tie-2: tyrosine kinase with immunoglobulin-like and EGF-like domains-2; COX-2: Cycloxygenase-2; mRNA: messenger ribonucleic acid; VEGFA: vascular endothelial growth factor A, VEGFR1/R2: vascular endothelial growth factor receptor ½; Tu: tumor; Adj: adjacent.

### Correlation of COX-2 gene expression and protein level with angiogenic factors in adjacent liver parenchyma

We found no significant correlation between COX-2 mRNA levels and any of the angiogenic factors in tumor-adjacent liver parenchyma, except for Ang-1 in adjacent cirrhotic liver (Table 2). None of the mRNA levels of the angiogenic factors in adjacent parenchyma correlated significantly with COX-2 protein.

### Correlation of COX-2 mRNA expression and COX-2 protein level

No correlation was found between COX-2 mRNA expression and COX-2 protein level neither in HCC tumor tissue nor in adjacent parenchyma (p>0.13). Calculation of the COX-2 protein:COX-2 mRNA ratio revealed a range from <1 to >35,000 in HCC tumor tissue and from 100 to >7,000 in liver parenchyma. These results suggest that post-transcriptional mechanisms are responsible for the highly variable COX-2 protein levels.

### MiR-16 expression in HCC and liver parenchyma and correlation with COX-2 expression

Figure 1C shows the relative expression of miR-16 in normal liver, tumor adjacent liver and in cirrhotic and noncirrhotic HCC tumor tissue. No statistically significant differences were found between any of these tissues. Also no correlation between miR-16 expression and either COX-2 mRNA or COX-2 protein was found.

### MiR-21 expression in HCC and liver parenchyma and correlation with angiogenic factors

Relative expression levels of miR-21 were higher in cirrhotic tissues –both in HCC tumor tissue and adjacent liver parenchyma- as compared to HCC and parenchyma in non-cirrhotic tissues (Figure 1D). Furthermore, HCC in cirrhotic liver revealed a higher expression of miR-21 versus adjacent liver parenchyma. Analyzing the correlation between miR-21 expression and the angiogenic factors revealed no correlations except for a negative correlation (r = −.76, p = 0.046) between miR-21 expression and the Tie-2 receptor in adjacent cirrhotic liver parenchyma.

### MiR-101 expression in HCC and liver parenchyma and correlation with Cox-2 expression

Relative expression levels of miR-101 were lower in adjacent liver parenchyma in cirrhotic liver as compared to adjacent parenchyma in non-cirrhotic liver (Figure 1E). MiR-101 expression did not correlate with COX-2 expression neither at the mRNA level nor at the protein level.

## Discussion

In this study we analyzed the expression of COX-2, miR-101 and miR-16 -both with COX-2 inhibiting effects- and miR-21 -with pro-angiogenic and pro-inflammatory effects- in relation to expression of various angiogenic factors in human HCC in cirrhotic and noncirrhotic liver. The chronic inflammation in cirrhotic liver provides a prosperous environment for development of HCC and because of the interaction between inflammation and angiogenesis we analysed the above mentioned molecules in two groups of patients; those with HCC in a cirrhotic liver and in patients with HCC in a noncirrhotic liver. The main finding of this study is that although at mRNA level COX-2 expression correlates positively with mRNA expression of the Ang-1/Tie-2 axis in both cirrhotic and noncirrhotic HCC, no such a correlation was encountered at the COX-2 protein level. As a possible explanation for this discrepancy we investigated the role of miR-101 and miR-16 in HCC tumor tissue and adjacent parenchyma. We found that neither miR-101 nor miR-16 had a correlation with COX-2 mRNA or COX-2 protein.

In prostate cancer cell lines it was demonstrated that miR-101 inhibits COX-2 by translational repression via binding to the 3′ untranslated region (UTR) of COX-2 mRNA [25]. In colon cancer cell lines a discrepancy was encountered between COX-2 protein levels and COX-2 mRNA expression; of six miRNAs examined only miR-101 demonstrated an inverse relation with the COX-2 protein/mRNA ratio in these cell lines [18]. The same authors investigated human primary colorectal cancer specimens and liver metastases and found the same inverse relation. In a series of 30 patients with gastric cancer more advanced tumor stages were associated with lower miR-101 expression which was inversely related to COX-2 mRNA expression [19]. All these data provide support for a possible role of miR-101 as a negative regulator of COX-2 protein expression and therefore we analyzed this interaction also in the HCC tumor tissue. However, we could not corroborate these findings since our data showed no inverse correlation between COX-2 and miR-101 in HCC.

MiR-21 is an important oncomir in many cancers and also in HCC tumor tissue [26], [27]. MiR-21 plays a role in angiogenesis as was demonstrated in experiments with cell lines as well as in human cancer [12], [13]. In prostate cancer cell lines inhibition of miR-21 was associated with a less aggressive phenotype via inhibition of tumor growth and invasiveness [28]. Of the miRNAs analysed in our study, the most prominent differences were encountered in miR-21 expression. MiR-21 revealed the highest expression in HCC in cirrhotic liver and also adjacent cirrhotic liver parenchyma showed a higher miR-21 expression as compared to noncirrhotic adjacenct liver parenchyma. This differential expression, however, did not translate into significant correlations with the studied angiogenic factors, except for a negative correlation with the Tie-2 receptor in liver parenchyma only. This adds support to the idea that clinically overt HCC –despite a different parenchymal background- share several molecular characteristics, which is in accordance with previous findings in which no differences were found between HCC in cirrhotic versus noncirrhotic livers with respect to endothelial cell turnover, microvessel density as well as expression of various angiogenic growth factors [29], [30]. MiR-21 is rather ubiquitous in health and disease [14], [31]. Therefore it is not surprising that upregulation of miR-21 has a pivotal role in many inflammatory processes, like in patients with chronic liver diseases in whom miR-21 serum levels correlated with the severity of liver fibrosis and necroinflammation [17], [32].

We found a discordance between COX-2 mRNA and protein level. As a possible explanation for this discrepancy post-transcriptional mechanisms, like transcript decay and translation rate, but also post-translational modifications which determine protein turnover and thereby protein expression levels have been described [33] [34]. In humans highly discrepant results were observed between mRNA and protein expression in a study of human lung adenocarcinomas [35]. Thus, mRNA abundance does not necessarily translate into the detection of the corresponding protein with the currently available techniques and conclusions should be drawn cautiously [23].

The lack of correlation between miR-16, miR-101 and COX-2 expression is in contrast to findings in other tumor types like gastric and colon cancer. We propose two possible explanations for this discrepancy, (1) insufficient expression level of the miRNAs and (2) (tumor)tissue specificity of the miRNAs. It is likely that the expression of miRNAs should be above a –as yet unknown- minimum threshold level before they can exert their function [36], [37]. Differences in miRNA expression might thus explain differences in functional actvivity. In addition, the overall abundance of all miRNA specific target genes in a given cell also may affect the targeting efficiency. For the analysis at the RNA level total tissue is used, and there might be a discrepancy between cells expressing the miRNA and the COX-2 gene. The current dogma describes that COX-1 is constitutively expressed and COX-2 is only expressed as a result of induction by a wide range of stimuli [38]. In an autopsy study on trauma victims, this concept was seriously questioned, because several healthy tissues, including the normal liver, were shown to express COX-2 [39]. We also found COX-2 expression in normal liver parenchyma; this was comparable to COX-2 expression in liver parenchyma adjacent to HCCs. In parenchyma adjacent to HCC we found that COX-2 expression was even higher than in HCC itself. Our data are in agreement with findings of Morinaga et al. who found a 2.5- fold higher COX-2 mRNA expression in adjacent liver parenchyma as compared to HCC [40]. In their study COX-2 protein expression -determined by immunohistochemistry- seemed to parallel mRNA expression and the authors explained this by inflammatory activity in tumor-adjacent parenchyma.

A limitation of our study is that the investigated tumor samples were obtained from clinically overt HCCs and the resulting signature will be rather different from the signature in dysplastic nodules and small –clinically undetectable- HCCs. For instance, COX-2 expression was found to be highest in dysplastic nodules with lower expression levels in more poorly differentiated advanced tumors in human HCC [41]. Another limitation of the study is the relatively low number of included patient samples.

From this study in human HCCs we conclude that COX-2 is expressed to a similar extent in cirrhotic and noncirrhotic adjacent liver parenchyma. Also no differences were found between HCC in cirrhotic and noncirrhotic liver despite the fact that COX-2 mRNA and COX-2 protein was lower in HCC in comparison to adjacent parenchyma. COX-2 mRNA levels both in cirrhotic and noncirrhotic HCC tumor tissue correlated with angiogenic factors. The inverse correlation between miR-101 and COX-2 protein –as found in colorectal cancer by others- was not found in our HCC tumor samples. The described prominent role of miR-21 as a pro-angiogenic miR could not be substantiated in this study. The clinical usefulness of COX-2 inhibitors especially in the setting of chemoprevention for the development of human HCC can only be based on randomized clinical trials since the precise regulatory mechanisms of COX-2 are far from elucidated.
